# Quantifying the contribution of chromatin dynamics to stochastic gene expression reveals long, locus-dependent periods between transcriptional bursts

**DOI:** 10.1186/1741-7007-11-15

**Published:** 2013-02-25

**Authors:** José Viñuelas, Gaël Kaneko, Antoine Coulon, Elodie Vallin, Valérie Morin, Camila Mejia-Pous, Jean-Jacques Kupiec, Guillaume Beslon, Olivier Gandrillon

**Affiliations:** 1 Université de Lyon, Université Lyon 1, Centre de Génétique et de Physiologie Moléculaire et Cellulaire (CGPhiMC), CNRS UMR5534, F-69622 Lyon, France; 2 Université de Lyon, INSA-Lyon, INRIA, Laboratoire d'InfoRmatique en Image et Systèmes d'information (LIRIS), CNRS UMR5205, F-69621 Lyon, France; 3 Laboratory of Biological Modeling, NIDDK, National Institutes of Health, Bethesda, MD 20892, USA; 4 INSERM, Centre Cavaillès, Ecole Normale Supérieure, F-75005 Paris, France

**Keywords:** Chromatin dynamics, expression noise, gene regulation, stochastic model

## Abstract

**Background:**

A number of studies have established that stochasticity in gene expression may play an important role in many biological phenomena. This therefore calls for further investigations to identify the molecular mechanisms at stake, in order to understand and manipulate cell-to-cell variability. In this work, we explored the role played by chromatin dynamics in the regulation of stochastic gene expression in higher eukaryotic cells.

**Results:**

For this purpose, we generated isogenic chicken-cell populations expressing a fluorescent reporter integrated in one copy per clone. Although the clones differed only in the genetic locus at which the reporter was inserted, they showed markedly different fluorescence distributions, revealing different levels of stochastic gene expression. Use of chromatin-modifying agents showed that direct manipulation of chromatin dynamics had a marked effect on the extent of stochastic gene expression. To better understand the molecular mechanism involved in these phenomena, we fitted these data to a two-state model describing the opening/closing process of the chromatin. We found that the differences between clones seemed to be due mainly to the duration of the closed state, and that the agents we used mainly seem to act on the opening probability.

**Conclusions:**

In this study, we report biological experiments combined with computational modeling, highlighting the importance of chromatin dynamics in stochastic gene expression. This work sheds a new light on the mechanisms of gene expression in higher eukaryotic cells, and argues in favor of relatively slow dynamics with long (hours to days) periods of quiet state.

## Background

Although the importance of stochasticity in gene expression has been anticipated more than three decades ago [1-3], the existence of a strong stochastic component in gene expression has only recently been experimentally demonstrated, showing that, despite constant environmental conditions, isogenic cells do show significant fluctuations in their gene-expression levels [4-10]. Moreover, regulated stochasticity, and its resulting phenotypic diversity, has been shown to be involved in several biological processes [11], including cell differentiation [12,13], development [14,15], virus decision-making [12,16], and bacterial survival during environmental stress [17-20].

Many studies have shown that the average expression level of a gene depends strongly on its genomic location [21-25]. In cultured cells, the silencing position effect (similar to the position effect variegation seen in *Drosophila *and mammals) is a well-characterized example of the influence of chromatin on gene expression; with a stably integrated transgene, a progressive silencing of the reporter occurs, at a rate that strongly depends on the integration site [26]. Several studies based on treatments with 5-azacytidine (a DNA-demethylating agent [27]) and with trichostatin A (a histone deacetylase inhibitor [28]) have shown that DNA methylation and histone acetylation play a pivotal role in this process. Indeed, these treatments reverse the extinction of the transgene [26,29]. Almost all of these studies, however, have focused on the mean value of gene expression, and only a few have addressed the question of the relationships between stochastic gene expression and chromatin, in either yeast [30-35] or higher eukaryotes [36-39].

Initially conducted in prokaryotes [4,40], experiments to explore the molecular causes of stochastic gene expression were rapidly extended to yeast models [6,31,41,42]. These experiments suggested that, other than trivial aspects such as small molecule numbers, more sophisticated causes, such as chromatin remodeling, were important players in stochastic gene expression [43]. More precisely, of the various possible sources of stochasticity, one in particular, namely locus-dependent chromatin dynamics (for example, transitions between an 'open' state that allows gene transcription and a 'closed' state that represses gene transcription) is a promising candidate to explain the regulation of stochastic gene expression. This role of chromatin was highlighted by the work of Becskei *et al*., who in 2005 showed the existence of genomic domains in the yeast genome, which produce a low transcriptional noise (that is, the part of stochastic gene expression arising from irregular transcript production) [31]. The following year, by analyzing the variability of mRNA levels from tandemly and non-tandemly integrated pairs of transgenes in mammalian cells, Raj *et al*. identified the influence of genomic domain on transcriptional noise, suggesting the importance of the switching rate between chromatin states via remodeling. Gene activation or inactivation would occur in cases of chromatin decondensation or condensation, respectively [36]. To analyze the effect of chromatin remodeling on promoter activation and therefore on stochastic gene expression, Raser and O'Shea used yeast strains lacking components of the chromatin-remodeling complexes. A major conclusion of their work was that the alteration of chromatin-remodeling enzymes resulted in changes in stochastic gene expression [42]. However, most of these studies have tried to link chromatin dynamics to stochastic gene expression using indirect approaches [31,36,42,44].

In many situations, from prokaryotes to eukaryotes, simple mathematical models describing the transcriptional dynamics as a two-state process have been shown to account effectively for the stochastic expression of a gene [45,46]. Indeed, the two-state model, also known as the 'random-telegraph model' [47,48], now constitutes a standard in the field. This model assumes that the promoter switches randomly between two states, 'on' and 'off', with only the former allowing initiation events to occur. These transitions could correspond to several mechanisms, including assembly and disassembly of specific complexes, progression through the cell cycle, or the recruitment of the locus into transcription factories [49]. In many cases, evidence supports the hypothesis that these 'on' and 'off' states primarily reflect alternative chromatin configurations [50].

Recently, using a short-lived luciferase protein, Suter *et al*. monitored transcription at high temporal resolution in single mammalian cells, and identified bursts of transcription, a mechanism previously suggested in prokaryotes and eukaryotes [4,36]. Using the random-telegraph model, they characterized the temporal patterns of transcriptional bursts for different genes, and obtained the distributions of the 'on' and 'off' times [51]. Harper *et al*. performed a complementary analysis of transcriptional bursting in single mammalian cells [52]. By quantifying the time dependence and cyclic behavior of the transcriptional pulses from the prolactin promoter, they estimated the length and variation of both transcriptionally active and inactive phases. Both studies point to the existence of a refractory 'off' period, but they diverge on the role of chromatin remodeling; in contrast to the Suter study, in which chromatin environment seemed to play a secondary role in shaping bursting patterns, Harper *et al*. concluded that chromatin remodeling may play an important role in the timing of transcriptional bursting. Finally, based on time-lapse fluorescence microscopy experiments, coupled with the use of the two-state model, Dar *et al*. gave a recent comprehensive study on noise in mammalian cells [53]. In their work, these authors suggested that transcriptional bursting, as opposed to constitutive expression, dominates across the human genome. Moreover, by analyzing more than 8,000 distinct genomic loci, they found that both frequency and burst size vary by chromosomal location. Therefore, the role of chromatin dynamics in the control of stochastic gene expression in higher eukaryotes remains a central matter of debate.

In a preliminary study, our group showed, using isogenic cell populations expressing a fluorescent reporter, that modification of chromatin marks, using chromatin-modifying agents such as 5-azacytidine (5-AzaC) and trichostatin A (TSA), induced significant effects on mean fluorescence intensity (MFI) and normalized variance (NV; that is, the variance normalized by the square of the mean) [11]. We also showed that TSA and 5-AzaC had different effects on NV, whereas their effects on MFI were similar. Finally, investigating the possible reversibility of the effects identified by flow cytometry after the drug treatments, we found that MFI, NV, and the shape of the fluorescence distributions tended to return to their initial values after the treatment end. This result, which shows full reversibility of the cellular system after important modifications of the chromatin state, suggests that cells could be able to temporally modify their level of stochastic gene expression via modifications of chromatin marks, before returning to their initial physiological state.

**Figure 1:**
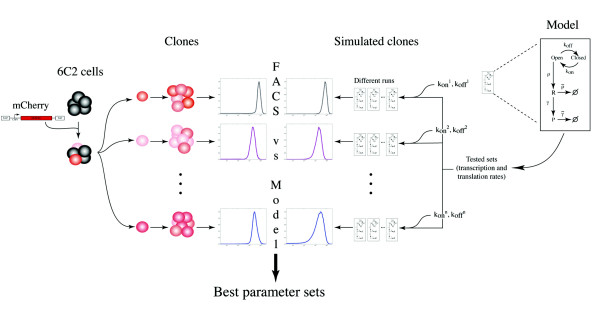
**Experimental strategy used for assessing the role of chromatin environment on stochastic gene expression**. After generation of cellular clones expressing the fluorescent reporter *mCherry*, stably integrated as a unique copy into the genome, the fluorescence distributions obtained by flow cytometry ('FACS') were compared with simulated distributions generated by a two-state model ('Model'). After experimental determination and exploration of transcription-translation parameters (*ρ*, transcription rate; *γ*, translation rate; $\tilde{\rho }$, mRNA degradation rate; $\tilde{\gamma }$, protein degradation rate and *α*, protein fluorescence coefficient), the best parameter sets were identified, and then used to compute the specific chromatin dynamics (*k_on _*and *k_off_*, which are, respectively, the opening and closing transition rates of the chromatin at the reporter integration site) for each clone.

To assess the possible influence of chromatin-opening/closing dynamics on the stochasticity of gene expression, the next step was to combine biological experiments with a modeling analysis. For that purpose, we generated a series of clonal isogenic cell populations from chicken erythrocyte progenitors (6C2 cells). These populations were stably transfected with a unique copy of a reporter-gene coding for the red fluorescent protein mCherry, but the reporter was inserted at different chromosomal positions in each clone (Figure 1, left). Using flow-cytometry measurements, we found substantial clone-to-clone differences in the stochastic expression of the reporter. In particular, some of the clones had very similar MFI but different NV values. Because the only difference between these clones was the genomic location of the reporter, the observed differences in stochastic gene expression must stem from the chromosomal positioning effect, such as locus-specific dynamics of the chromatin surrounding the transgene. To evaluate whether chromatin dynamics significantly affect the stochasticity of gene expression, we treated some clones with 5-AzaC and TSA. Cell responses to these drugs clearly showed that both MFI and NV were affected, indicating that the chromatin environment of the reporter gene plays a significant role in the stochasticity of its expression. This result confirmed preliminary conclusions obtained by our team [11]. By fitting a two-state model to the experimental data, we provided a mechanistic interpretation for the clone-to-clone diversity of expression patterns, in terms of differences in chromatin dynamics. More specifically, based on both analytical derivations [45] and simulations [54], we explored the dynamics of the model and iteratively refined its kinetic parameters. The outcome was an accurate reproduction of the distribution of expression levels before, during, and after drug treatment.

Our current study supports the view that expression dynamics is strongly driven by short and infrequent transcriptional bursts, as previously described in other models, including mammalian models. However, the major advance of this work is that, whereas the duration and intensity of bursts did not show strong clone-to-clone differences, the time between bursts was found to depend strongly on genomic location and was broadly affected by drug treatments that affect chromatin. Hence, the position-dependent opening dynamics of chromatin emerges as a key determinant of the stochasticity in gene expression.

## Results

**Figure 2:**
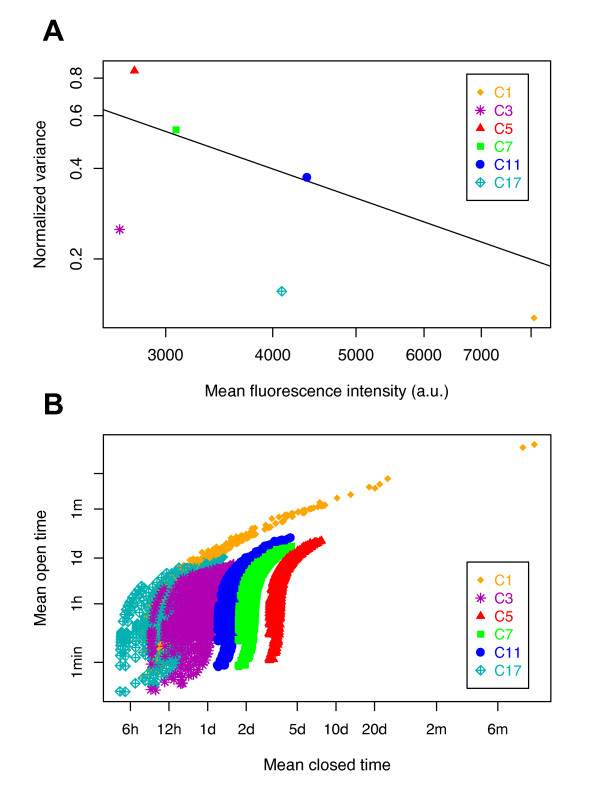
**Exploration of model parameters to explain the observed stochastic gene expression for six cellular clones**. **(A) **Relationship between normalized variance (NV) and mean fluorescence intensity (MFI) for six cellular clones (C1 to C17) stably transfected with a unique copy of the fluorescent reporter *mCherry *that was integrated at a different locus in each clone. Black line shows the relationship NV = 1/MFI. **(B) **Distributions of the possible chromatin dynamics. For each clone, all 1,087 possible couples of (1/*k_on_*; 1/*k_off_*) values were plotted, expressed as mean open time (1/*k_off_*) and mean closed time (1/*k_on_*) for all transcription-translation parameter sets explored analytically in the two-state model (see Methods). One dot therefore represents one possible analytical solution for that clone. h, hours; d, days; m, months.

We generated a series of clones stably transfected with the *mCherry *reporter, driven by the cytomegalovirus (CMV) promoter, then using splinkerette PCR [55], we retained six clones showing a unique reporter insertion site (see Additional file 1, Table S1). These clones were then analyzed by flow cytometry, yielding for each of them the full distribution of fluorescence, and the corresponding MFI and NV (Figure 2A). It is important to emphasize that the six clones differed only in their reporter insertion sites. Based on the NV, a robust indicator of the stochasticity of gene expression [56], the clones could be sorted from the most to the least stochastic, in terms of reporter-gene expression as follows: C5>C7>C11>C3>C17>C1. Moreover, analyzing the relationship between NV and MFI, we concluded that there is no direct linear relation between these two parameters. Indeed, certain clones displayed similar MFI but very different NV values (for example, comparison of C3 with C5, or C11 with C17, Figure 2A). This important dispersion of the points, around the inverse tendency between NV and MFI values, also suggests that mRNA abundance fluctuations were not the major source of intrinsic noise in this context.

An explanation of these observations comes from a previous preliminary study, in which we investigated whether chromatin dynamics are involved in these observed differences [11]. Using the same cellular clones (same cell line, reporter, and environmental conditions) we performed the 5-AzaC and TSA treatments that would act directly on chromatin by two different molecular means. Our results showed that for the two drugs, modification of chromatin dynamics had clear consequences for stochastic gene expression [11]. However, in this previous study, we did not assess how chromatin influences stochastic gene expression.

Thus, for this purpose in the current study, we fitted these data to a two-state model of gene expression, and evaluated to what extent chromatin dynamics act on stochastic gene expression. Under the assumption that all parameters but those describing the dynamics of chromatin would be identical in all the clones, we performed an iterative screening of model parameters. This allowed us to find these common parameters, and to characterize the position-specific dynamics of chromatin for each individual clone (Figure 1).

### Description of the model

The choice of the model used to analyze our biological data was crucial. Two models are classically used to describe transcriptional stochasticity: 1) a Poisson model, in which the gene has, at each instant, a given chance to produce an mRNA, [7,47,57] and 2) a random-telegraph model, in which the gene additionally switches randomly between an 'on' state, in which transcripts are produces in line with Poisson dynamics, and an 'off' state, in which no transcripts are produced [43,45,57]. The Poisson model is known to lead to a direct linear relationship between MFI and NV on a log-log plot (that is, NV = 1/MFI) [38,58]. Because such a relation was not sufficient to describe our data (Figure 2A), we adopted the more general random-telegraph model. It cannot be excluded that extrinsic noise may also participate to some degree in the observed fluorescence distributions. However, observing a variety of distributions for different insertion sites of the reporter (Figure 2A) strongly suggests that the major source of noise is intrinsic. Indeed, as sources of extrinsic noise are independent of the reporter, they were expected to have somewhat similar effects in all the different clones. In addition, given the long mRNA and protein lifetimes in our system (see below), only the very slow extrinsic fluctuations are likely to affect the protein levels of the reporter.

Because flow cytometry quantifies protein fluorescence, the model must describe the expression process up to the protein level (including mRNA and protein production and degradation rates) and requires an additional parameter to convert protein quantity into fluorescence intensity (Figure 1, right). Thus, for each clone, the model had seven parameters: *k_on _*and *k_off _*, respectively describing the rates of chromatin opening and closing, *ρ *and *γ*, describing the transcription and translation rates, $\tilde{\rho }$ and $\tilde{\gamma }$, describing the transcript and protein degradation rates, and finally, a linear coefficient *α*, representing the fluorescence intensity of a single mCherry protein in the arbitrary unit measured by the flow cytometer. In order to fit the model, the optimal set of parameters must be identified, under the assumption that *ρ*, *γ*, $\tilde{\rho }$, $\tilde{\gamma }$ and *α *are identical in every clone, but that *k_on _*and *k_off _* are clone-specific. From this point, we refer to the five former parameters as the 'transcription-translation parameters' and to the two latter ones as the 'chromatin-dynamics parameters'. Because we had six clones, we actually had to determine 17 parameters ((6 × 2) + 5) in order to fully specify the model and to ultimately estimate the chromatin-dynamics parameters for each clone. For these 17 parameters, the two degradation rates ($\tilde{\rho }$ and $\tilde{\gamma }$) were determined experimentally from inhibition-based experiments (see Methods; see Additional file 2, Figure S1). We found respectively that $\tilde{\rho }$ = 1.63 × 10^-3^/min (mRNA half-life of 7 hours and 4 minutes) and $\tilde{\gamma }$ = 1.76 × 10^-4^/min (protein half-life of 65 hours and 47 minutes). The sensitivity of our results with regard to uncertainty in these experimentally determined values will be discussed later. These values are consistent with average mRNA and protein half-lives previously measured in mammalian cells (9 and 46 hours, respectively) [59]. Following this, we needed to find the optimal values of a set of 15 parameters to fit the experimentally measured fluorescence distribution of the six clones.

Several methods can be used to find such a parameter set. In particular, there are various optimization methods available, such as simulated annealing. However, because the model-experiment comparisons in our study involved stochastic simulations, the objective functions that have to be minimized (that is, some distance measure between predictions and observations) are only estimated up to a certain error level. Although small, this error level makes most optimization algorithms inadequate. Indeed, these algorithms rely on estimating the gradient or Hessian of the objective function, based on a finite difference procedure (that is, evaluating small variations in the objective function resulting from small variations in its parameters). In a context where successive estimations of the objective function, even for the same parameters, may display random variations, these optimization algorithms are clearly doomed to failure. Overcoming this issue would require both running extremely long and computationally intensive simulations to minimize the error, and using coarse variation steps in the gradient-estimation procedure, which could result in numerical instabilities during the optimization.

For this reason, we decided to conduct a systematic parametric exploration, as this is a procedure that does not require local smoothness of the objective function. In addition, a single evaluation of the objective function represents a heavy computation load; for example, involving thousands of realizations of a Gillespie simulation that are followed over long periods of simulated time (see Methods). In this context, a systematic parametric exploration allows massive parallelization of the computations on a grid. The sequential evaluation imposed by optimization algorithms makes this approach prohibitive. However, because the systematic exploration still requires intensive computations, we used iterative screening of the model parameters to progressively reduce the parameter space that has to be simulated.

This iterative screening was based on three steps in which we successively used analytical derivations on the model (step 1), additional experimental data (step 2), and finally, stochastic simulation (step 3). Thanks to these successive screenings, we were able to reduce by a factor of 30 the number of parameter sets to be simulated, thus making the problem computationally tractable. In the following sections, we describe the three screening steps and the results we obtained from them.

### First screening of model parameters, based on mean and variance of fluorescence intensity

Mathematical derivations by Paulsson from the two-state model [45] analytically provided the values of MFI and NV as a function of all parameters: *k_on_*, *k_off _*, *ρ*, *γ*, $\tilde{\rho }$, $\tilde{\gamma }$ and *α*. By inverting these equations (see Methods), we were able to compute the chromatin-dynamics parameters (*k_on _*and *k_off_*) for each clone from: 1) the experimentally measured MFI and NV of the clone, 2), the experimentally determined values of $\tilde{\rho }$ and $\tilde{\gamma }$, and 3) the unknown transcription-translation parameters (*ρ*, *γ *and *α*). Thus, only three transcription-translation parameters remained to be determined, making their combinatorial exploration computationally tractable.

We explored wide ranges of these parameters that included all biologically relevant values [60]: 20 values for *ρ *(from 6 to 0.00833 mRNA/min; that is, a transcription event occurring from every 10 seconds to every 2 hours when the chromatin is open), 15 values for *γ *(from 1 to 0.0003472 protein/min/mRNA; that is, a translation event occurring from every 1 minute to every 2 days for each mRNA), and 12 values for *α *(from 0.1 to 200 fluorescence units per protein) [61] (see Methods for the exact tested values). For each triplet (*ρ*, *γ*, and *α*), we computed *k_on _*and *k_off _*for each of the six clones from their experimental values of MFI and NV. Of the 3,600 initial parameter sets, only 1,087 led to valid solutions, with the others leading to negative values for *k_on _*or *k_off _*for at least one clone. Figure 2B shows the 1,087 possible pairs of values (*k_on_*; *k_off_*) that resulted from this exploration for all the clones. It was found that, although the chromatin-dynamics parameters could be the same order of magnitude, the mean open time (1/*k_off_*, roughly between 1 minute and 1 day) was markedly shorter than the mean closed time (1/*k_on_*, roughly between 6 hours and 4 days). This is characteristic of a transcriptional activity in which mRNA production events occur in brief bursts separated by longer silent periods.

The result of this first screening still produced more than 1,000 valid parameter sets, with the values of *k_on _*and *k_off _*spanning large intervals. This emphasizes that NV and MFI alone are not sufficient to identify, for a specific clone and therefore for a given genomic insertion site, the parameters that best explain the observed distribution of fluorescence.

### Second screening of model parameters, based on response to treatments with chromatin-modifying agents

**Figure 3:**
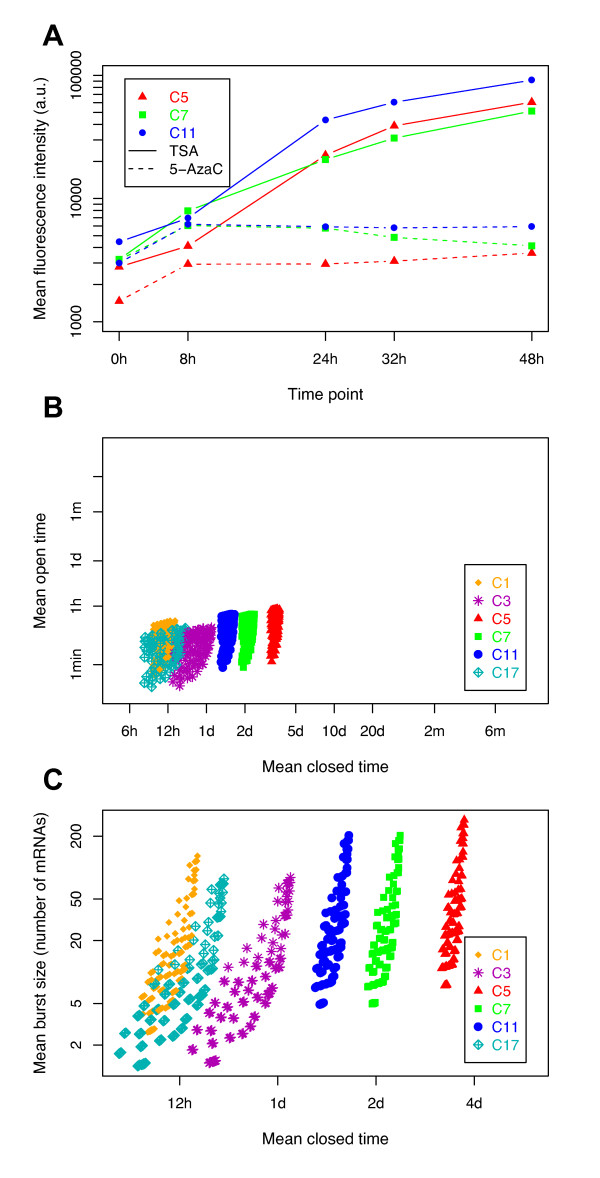
**Exploration of model parameters based on treatments with chromatin-modifying agents**. **(A) **Evolution of mean fluorescence intensity following kinetics of treatment with trichostatin A (TSA; solid line) and 5-azacytidine (5-AzaC; dotted line) (0 to 48 hours) for three cellular clones. **(B) **Distributions of the plausible chromatin dynamics. For each clone, all 114 possible couples of (1/*k_on_*; 1/*k_off_*) values were plotted, expressed as mean open time (1/*k_off_*) and mean closed time (1/*k_on_*), after removal of all parameter sets that were not able to account for the transcription-translation dynamics under TSA and 5-AzaC treatments. **(C) **This experiment was the same as for (B), except than the transcription rate (*ρ*) and the mean open time (1/*k_off_*) parameters were reduced to a single effective parameter (*ρ*/*k_off_*), representing the mean burst size. min, minutes; h, hours; d, days; m, months.

In order to reduce the ranges of solutions, we conducted additional experiments in which we modified the global dynamics of chromatin in both the cells and the model. We first treated three clones with the two chromatin-modifying agents TSA and 5-AzaC. As expected, TSA treatment, which leads to chromatin decondensation [62,63], induced an increase in MFI over time (Figure 3A). 5-AzaC treatment, which inhibits chromatin condensation [64], produced the same effect as TSA treatment, but to a much lower extent. It is noteworthy that measures such as MFI, NV, and the fluorescence distributions tended to return to their initial values after removal of TSA and 5-AzaC, indicating full reversibility of the cellular system, and therefore a conservation of physiological conditions [11].

Based on these additional data, we could then exclude all transcription-translation parameter sets that did not account for the observed increase in expression levels even if the chromatin was considered as constantly open (see Methods). It is important to emphasize that we made the assumption that the TSA and 5-AzaC treatments affected only the chromatin-dynamics parameters. Using this strategy, we were able to reject 86% of the parameter sets, thus we kept only 114 transcription-translation parameter sets for further analyses. Figure 3B shows the chromatin-dynamics parameter sets (that is, *k_on _*and *k_off_*), corresponding to the transcription-translation sets that were kept. All retained cases had in common that the mean open time 1/*k_off _*was very short compared with any other timescale in the model (in particular both the mean closed time 1/*k_on _*and the mean mRNA lifetime $1/\tilde{\rho }$). Hence, the actual duration of the bursts could not be estimated because two parameter sets with different *k_off _* but an identical number of mRNAs produced per active period will exhibit similar distributions. For instance, if, on average, 20 mRNAs are produced during bursts that last 30 seconds or during bursts that last 10 minutes, the results will be practically identical because mRNAs decay with a half-life of more than 7 hours. Hence, as in other studies [36], we could not determine the parameters *k_off _* and *ρ*, but only their ratio *ρ*/*k_off _*, that is, the mean number of mRNAs produced during a burst. This new effective parameter, referred to as 'burst size', reduces by 1 the number of parameters in the model. At a higher level, protein synthesis/degradation noise is only important in cases where there is a low copy number [45]. Because low protein abundance would not be detected in a cytometry measurement, this source of noise is marginal compared with the noise from transcription and mRNA synthesis/degradation. For instance, even for the least variable clone (C1, which had NV = 0.12 approximately, in Figure 2A), a mean protein level as low as 200 copies would only contribute less than 5% to the measured NV. Hence, the parameters *γ *and *α *directly compensate for each other, and can be grouped into a single effective parameter, '*α*·*γ*' (for example, producing twice as many proteins with half the fluorescence does not affect the distribution of fluorescence), reducing again by 1 the number of fitting parameters.

Reformulating the sets of (1/*k_off _*; 1/*k_on_*) couples retained after the second screening (Figure 3B) in terms of (*ρ*/*k_off_*; 1/*k_on_*), as shown in Figure 3C, we observed relatively similar ranges of values for the mean burst size *ρ*/*k_off _*for the six clones (although values spanned from 1 to 200 mRNAs per burst). By contrast, the mean closed time of chromatin seemed to be highly clone-dependent, ranging from 6 to 12 hours for clone C1 and C17 to more than 2 days for C5 and C7 (Figure 3C). This suggests that the chromatin-opening dynamics depend on the clones, and therefore on the chromatin environment of the reporter.

### Third screening of model parameters, based on full distribution of fluorescence

To select the best parameter set from the 114 remaining sets, we simulated distributions of fluorescence corresponding to the remaining parameter sets, and compared them with the fluorescence distributions measured by flow cytometry. For each parameter set, we used a stochastic simulation algorithm (SSA) [54], to simulate 50,000 cells per clone, and then computed the resulting fluorescence distributions. Background fluorescence levels were added to the simulated distributions by convolution with the fluorescence distribution of the negative control-cell population (that is, cells that did not express any fluorescent protein). The resulting values were then compared with the six experimental distributions using a Kolmogorov-Smirnov test.

**Figure 4:**
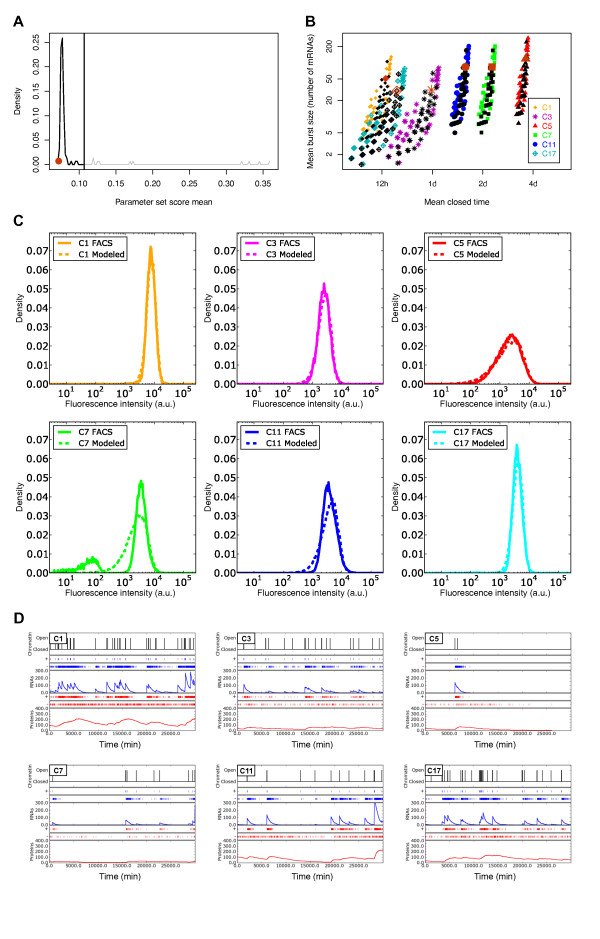
**Exploration of model parameters based on a comparison of fluorescence distributions and stochastic simulation algorithm (SSA) simulations**. **(A) **Distribution of parameter set scores. The lowest scores correspond to the better fits. These fits were obtained using values of *γ *and *α*, the parameters contained within the joint *α*·*γ *value of 0.035 arbitrary unit/min/mRNA. The upper limit (0.107) of the single peak showing the best scores is specified (vertical line). **(B) **Distribution of chromatin dynamics ('mean burst size' and 'mean closed time'), obtained for the best parameter sets, after distribution comparisons for the six cellular clones. To compare with the possible chromatin dynamics presented in Figure 3B, this figure shows the chromatin dynamics obtained for the best parameter sets (black; score means between 0.07 and 0.107; see panel (A)) and the optimal parameter set for each clone (brown). **(C) **Illustration, for the six cellular clones, of the comparison between the mCherry fluorescence distributions measured by flow cytometry ('FACS'; solid line), and simulated fluorescence distributions ('Modeled'; dotted line) obtained with the best chromatin-dynamics parameter set. **(D) **One run of Gillespie SSA per clone showing the chromatin dynamics (opening and closing chromatin events are shown in black) for one virtual cell of the isogenic population distribution (see panel (C)). Consequences of chromatin open/closed dynamics on mRNA transcription and protein translation are shown in blue and in red respectively. Production (+) and degradation (-) evolutions of mRNAs and proteins are also indicated. (For illustration, Figure S2 (see Additional file 3) shows the same analysis as that presented in this figure, but for the parameter set with the highest (that is, worst) comparison score among the best ones).

Analyzing the comparison scores (distances) from the Kolmogorov-Smirnov test of the 114 parameter sets, we were able to identify the subsets of parameters, and therefore the corresponding chromatin dynamics, that were the best fit to the distributions measured by the flow cytometer (Figure 4A). Note that most sets correctly fit the experimental data (104 of the 114 sets corresponding to a single peak of good scores; that is, <0.107), showing that the previous screening had already selected the correct parameter sets. The final parameter sets are shown (in black) in Figure 4B. For five of the six clones, we were able to generate distributions similar to those measured by flow cytometry (Figure 4C). However, when analyzing the bi-modal clone C7, we found that the simulated distribution fit only the high modality of the fluorescence distribution. This third screening supports our previous observation about the relatively similar mean burst size between the clones but the significantly different mean closed times (Figure 4B). Looking at the chromatin-dynamics parameter set that best fit the flow-cytometry distributions for all clones (Figure 4B, in brown), our study revealed that for the six clones, mean burst sizes were between 30.0 and 118.9 mRNAs per burst, and mean closed times between 756.7 minutes (~12 hours) for the fastest clone to 5197.6 minutes (~3.5 days) for the slowest clone. However, it is important to note that, taking into account the full range of viable parameters (Figure 4B, in black) clone dynamics could be fit with similar values for their mean burst sizes (ranges of correct values are overlapping between the clones) whereas their mean closed time had to be different (Figure 4B).

Figure 4D illustrates, for each clone, the results of simulations of the chromatin dynamics of a single cell, for the best parameter set. The best chromatin-dynamics parameters for each of the six clones are shown in Table 1. It is interesting to compare the differences between the different clones (that is, for the different chromatin environments) in terms of chromatin dynamics and their consequences on the transcription and translation of the *mCherry *reporter. It seems clear that the transcriptional activity of the reporter can vary from frequent bursts (C1) to rare bursts (C5), depending on the chromatin context. These important differences could very well be the dependence of the local chromatin properties at the reporter insertion site. Finally, the mRNA transcription rates and mRNA copies per cell we defined for the six clones (on average 2.1 and 21 respectively) (see Additional file 4, Table S2) were in the same order of magnitude as those previously reported [59,65].

**Table 1:** Chromatin-dynamics parameters proposed for the six cellular clones.

Clone	1/*k_on_*^a^	*ρ*/*k_off_*^b^
C1	756.7	50.9
C3	1420.5	31.2
C5	5197.6	118.9
C7	3267.7	83.6
C11	2271.8	82.8
C17	882.7	30.0

^a^Mean closed times (1/*k_on_*) are expressed as minutes.

^b^Mean burst sizes (*ρ*/*k_off_*) are expressed as mRNAs.

### Chromatin dynamics at genomic insertion sites and sensitivity analysis

**Figure 5:**
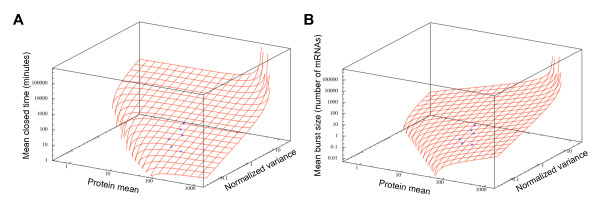
**Inference of burst size and closed time from mean and normalized variance (NV) of protein levels**. **(A) **At steady states, using the best transcription-translation parameter set (*ρ*, $\tilde{\rho }$, *γ*, $\tilde{\gamma }$ and *α*) and the modified Paulsson's equation system, the mean closed time could be calculated from the protein mean and protein NV (red grid). **(B) **Using the same data and equation system as in panel (A), the mean burst size could be calculated from the protein mean and protein normalized variance (red grid). Note that grids of both panels are linked because each value pair (protein mean and NV) corresponds to a single value pair (mean burst size and mean closed time). For both parts, clones C1, C3, C5, C7, C11, and C17 are represented as blue points on the grid, and all axes are on a logarithmic scale.

By combining biological experiments, analytical computations and stochastic simulations, we were able to estimate all the model parameters that best fit the measured flow-cytometry distribution for the different integration sites. We now used some of these parameters (that is, *α*·*γ*, $\tilde{\rho }$, and $\tilde{\gamma }$) to directly estimate the possible chromatin-dynamics parameters for any couple (MFI and NV), each corresponding to a different genomic insertion site of the reporter. We also used these parameters to estimate the sensitivity of the model (that is, the variation in the chromatin-dynamics parameters depending on the two main indicators of gene expression, MFI and NV) in a biologically relevant parameter space. Indeed, we were able to use the best set of transcription-translation parameters that we obtained, along with a modified Paulsson's equation system, to determine the mean closed time of chromatin and the mean size of transcriptional bursts from the mean and NV of any similar construction (that is, the same cells but different insertion point) measured by flow cytometry (Figure 5). This can be represented by two three-dimensional graphs: one for the mean closed time and one for the mean burst size. It should be noted that both graphs are linked because each couple (MFI and NV) corresponded to a single couple (mean burst size and mean closed time). Two important elements could be derived from these three-dimensional graphs. First, as shown in panel A, the mean closed time was determined mainly by the NV value, whereas MFI only had a marginal contribution (at least in the activity domain of the measured clones). In other words, whatever the average transcriptional activity, the mean closed time could be derived directly from the variability in expression levels, highlighting the informational content of stochasticity in gene expression [66]. By contrast, it can be seen from panel B that, to compute the mean burst size, both measures are necessary. Interestingly, the results presented here show that, for our cell lineage, fluorescence distributions, which are relatively easy to measure by flow cytometry, coupled with a pertinent and robust analysis, allowed us to obtain valuable information about the chromatin-dynamics parameters.

Finally, we determined how the reported values (Table 1) are affected by uncertainty in the experimentally determined mRNA and protein half-lives by conducting sensitivity analysis on equation 3 (see Methods). We found that variations of ±5% of either mRNA or protein half-life resulted in variations in mean closed time and mean burst size that were always smaller than 5%. We therefore concluded that any experimental uncertainty in the mRNA and protein half-lives would only marginally affect the parameter values obtained through the model.

### Testing and validation of the model following a dynamic evolution of the chromatin state

**Figure 6:**
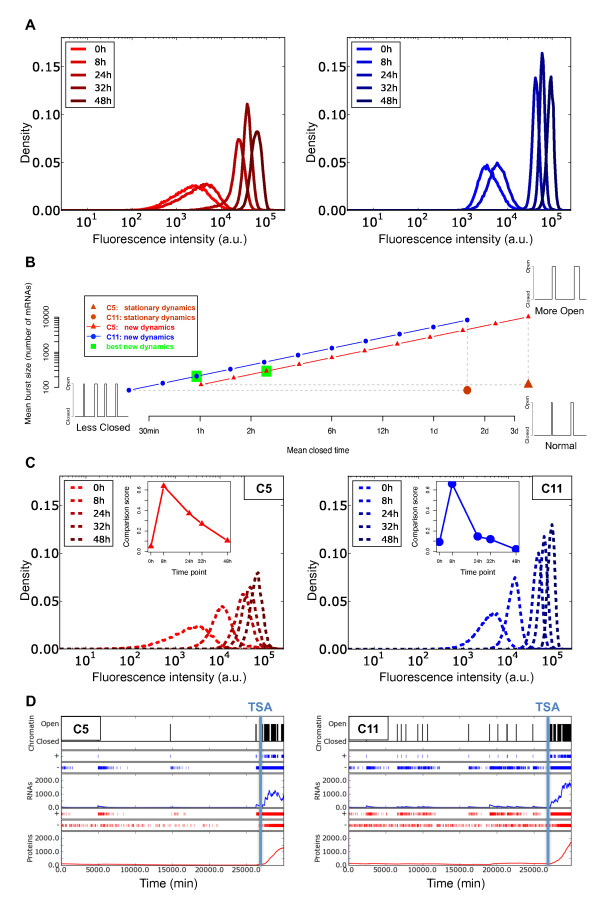
**Model simulation of the perturbation of chromatin dynamics after trichostatin A (TSA) treatment**. **(A) **Effects of TSA-treatment kinetics on the mCherry fluorescence distributions for two cellular clones, C5 (red) and C11 (blue) measured by flow cytometry. **(B) **New chromatin dynamics (mean burst size (*ρ*/*k_off_*) and mean closed time (1/*k_on_*)) fitting the observed fluorescence distribution evolution induced by TSA treatment. Different examples of these chromatin dynamics, inducing a higher open mean time (resulting from TSA treatment), are illustrated in the detailed view. After distribution-comparison tests, the best new chromatin dynamics (green), and those related to the steady state (brown) were ascertained. min, minutes; h, hours; d, days. **(C) **Simulated mCherry fluorescence distribution evolution obtained for the best new chromatin dynamics (see panel (B)). (Insets) Evolutions of the distribution-comparison scores (comparisons between measured distributions after TSA treatment and the simulated distributions). **(D) **One run of the Gillespie SSA per clone showing the dynamics of the chromatin before and during 48 hours of TSA treatment (opening and closing chromatin events are shown in black) for one virtual cell of the isogenic population distributions (see panel (C)). Consequences of chromatin open/closed dynamics on mRNA transcription and protein translation are shown in blue and in red respectively. Production (+) and degradation (-) evolutions of mRNAs and proteins are also shown. The beginning of TSA treatment is indicated by a vertical blue line. (For illustration, Figure S3 (see Additional file 5) shows the same analysis as presented in this figure but for a parameter set (same as used in Additional file 3, Figure S2) showing a weaker fit).

To test the contribution of chromatin dynamics to stochastic gene expression and the quality of the parameter set we obtained, we used our model to simulate a situation in which the chromatin dynamics were profoundly modified. For this, we used the flow-cytometry data from the TSA-treated clones C5 and C11 (5-AzaC was not tested because it produced less intense effects). During TSA treatment, the distributions of fluorescence, reflecting the expression of the *mCherry *reporter, gradually shifted to higher fluorescence values (Figure 3A, 6A). According to our study, to obtain such dramatic effects, the dynamics of chromatin at the reporter insertion locus must have been modified by reducing the mean closed time, increasing the mean burst size, or a combination of both. Because both parameters affect the transcriptional activity of the reporter, all the possible combinations that can account for the observed change in expression form a line in the mean closed time/mean burst size space (Figure 6B; see Methods). We explored the chromatin dynamics for parameter sets lying along this line, and found the set that best fit the new flow-cytometry data. As for the previous experiments, we systematically explored the different parameter sets by sampling 11 points on the line between the two extreme situations mentioned above. It should be noted that, for this exploration, we considered the transcription-translation parameter set as constant, identical to the one computed previously (Figure 4A). We found that the TSA treatment seems mainly to modify the chromatin mean closed time; for the two clones used in this experiment, TSA reduced the mean closed time from more than 1 day (C11) and more than 3 days (C5) to 1 and 2.5 hours respectively (Figure 6B). By contrast, mean burst size seemed to be increased only slightly. To support this result, we performed stochastic simulations with the retained chromatin-dynamics parameters to generate fluorescence distributions that we compared with the experimental flow-cytometry distributions (Figure 6C). For the two clones, the simulated distributions correctly fit the flow-cytometry values at the end of the TSA treatment (48 hours). However, for the first time point (8 hours of treatment), the simulated fluorescence distribution was shifted relative to the biological experiment.

The evolution of the comparison score between the measured and simulated data (Figure 6C, insets) confirmed that during the first hours of treatment the simulation was a poor fit to the flow-cytometry data. However, after 24 hours, a significant improvement occurred, and after 48 hours of treatment, the scores measured for the two clones were equivalent to those measured before the TSA treatment (0 hour of treatment, Figure 4A, C). This clearly demonstrates that the model correctly rendered the new chromatin dynamics at steady state, although it was not able to fully reproduce the transient period. This is probably due to the kinetics of the drug effect, which was considered immediate in the model (the chromatin dynamics being changed immediately at the treatment time) whereas, in real cells, the chromatin modifications probably take place more gradually, thus delaying the activity of the drug.

To illustrate the consequences of the new chromatin dynamics on the transcription and translation induced by the TSA treatment, Figure 6D shows SSA simulations of the cell dynamics for the two clones before and after treatment. Owing to the low frequency of chromatin-opening events before treatment, a period of more than 10 days is shown whereas the TSA treatment was simulated for only 48 hours. The simulation clearly indicates the effects of TSA treatment on the chromatin dynamics and emphasizes the increased frequency of the chromatin-opening events, resulting in an increase in mRNA and protein concentrations.

## Discussion

The importance of stochasticity of gene expression in many key cellular activities was appreciated many decades ago, and is now supported by strong experimental evidence [11].

Analyzing stochastic expression of a stably integrated fluorescent reporter in six isogenic cell populations, differing only in their reporter integration site, this study provides new evidence suggesting that the local chromatin environment (reporter insertion site) influences stochastic gene expression. Our results are in agreement with previous studies on HIV gene expression, where it was shown that the existence of different fates for infected cells correlated with the virus-integration sites [16,50], and that transcriptional burst size and burst frequency vary depending on the virus-integration sites [38,39]. This chromosomal positioning effect on stochastic gene expression was also shown in yeast and in mammalian cells [31,36], suggesting the existence of genomic local domain-level noise, probably under the control of the switching rate of chromatin between the open and closed configurations [31,36,42]. The biological function of this domain-level noise is not yet completely understood. Batada and Hurst showed in yeast that genomic domains that enable low noise act as sinks for essential genes, for which noise is more deleterious than for nonessential ones [67], suggesting an evolutionary pressure for shaping low-noise genomic domains. It is to be noted here that local chromatin dynamics is not the sole difference between the integration sites; other genomic features, possibly correlated with chromatin states, could also be involved. We are currently investigating such a question on a genome-wide scale.

Using a two-state model, we found that the observed NVs and MFIs for each clone alone are not sufficient to identify efficiently, for a specific chromatin environment, a restricted set of parameters that best explain the observed differences between the six clones. We thus used a more complex strategy exploiting the full distribution of fluorescence as measured by flow cytometry. By mixing analytical models, complementary experiments, and stochastic simulations, we progressively identified the parameters that best fit the flow-cytometry distributions. The final set of parameters we obtained was able to reproduce accurately the experimental data for all clones except the unique bi-modal one, C7, for which the simulated distribution fit only the high modality. This bi-modal distribution observed for clone C7 could be due to: 1) specific chromatin dynamics related to the genomic insertion site of the reporter, or 2) a genetic mutation event affecting the reporter-gene integrity and resulting in two genetically distinct subpopulations. In the first case, if the transition rates between active and inactive states are extremely slow relative to transcript and protein degradations, each promoter state would be relatively stable, and this transcription regime could result in bi-modal protein expression [4-6]. However, in the context of a two-state model, the value of the distribution between the two modes normally reflects the transient dynamics taking place after the gene switches from one state to the other, producing distribution tails from each mode towards the other. For clone C7, this part of the distribution between the two well-separated modes was notably low, almost null. This indicates that the passage from one state to the other was extremely rare, so rare that the protein half-life (although rather long at ~66 hours) is negligible in comparison. Such a slow dynamic is unlikely to be caused by chromatin dynamics. Indeed, during the submission of this work, very recent experimental evidence suggested that the bi-modal distribution of clone C7 arose from a genetic mutation of the reporter (Dr Alexander Skupin, Dr Aymeric Fouquier d'Herouel and Dr Sui Huang, ISB, personal communication). This event induces extinction of the transgene in a subpopulation of C7, and the appearance of the low modality (data not shown). Including this subpopulation in the fitting process would therefore induce a bias. Consequently, we re-ran the analysis, taking into account only the five clones showing a unimodal distribution. The chromatin-dynamics parameter set that best fit the flow-cytometry distributions for all clones presented in Figure 4B remained identical (data not shown), in accordance with the fact that the initial fitting process fit only the high modality of C7 and ignored the low one.

After selection of the best parameter sets and characterization of the chromatin dynamics for each clone, our work provided elements suggesting that the chromatin state is essentially dominated by the closed state, as previously shown [52], but most importantly, that the chromatin environments of the clones clearly differed in their mean closed time. Indeed, for all clones, the mean burst sizes roughly comprised between 30 and 120 mRNAs per burst, which is consistent with previous quantifications [36,51,53,68-70], whereas the means closed times were much more markedly clone-specific (roughly distributed between 12 hours and 3.5 days). This result suggests that the duration of the chromatin closed state could explain the basal stochastic gene expression differences observed between the six clones, in contrast to the mean burst size, for which values overlapped when considering all the best parameter sets. Therefore, the mean closed time could be an essential relevant parameter involved in the regulation of stochastic gene expression. The simulation demonstrated the existence of a highly bursty transcription process. It is noteworthy that a previous study using the CMV promoter did not observe such transcriptional bursts or intervals of inactivity [71]; however, that study used timescale analysis with a window that was significantly shorter than that used in our work. The use of the CMV promoter was essential for our study. In addition to overcome technical bias (see Methods), the fact that, using a strong promoter, we found significant differences between clones in gene-expression dynamics, and therefore genomic-integration sites, suggests that the source of the observed noise is related to the gene context (for example, chromatin state). The study strongly suggests that similar results could be obtained using a weaker endogenous promoter. Recent literature seems to corroborate this hypothesis; in the recent work of Dar *et al*., the authors showed that the genomic-integration site influences burst kinetics, with a the promoter type having a marginal influence [53]. Understanding promoter-specific effects would require abolishing the context effect that is, performing a study using different promoters in a controlled genomic location. This is currently being addressed in our group.

The results presented here also show how, using a two-state model and fluorescence distributions measured by flow cytometry, possible chromatin-dynamics parameters can be identified. In this study, the filtering of promoter activity by mRNA and protein dynamics allows inference of temporal information from a steady-state measurement (that is, fluorescence distributions). In this regard, the mRNA and protein half-lives are the components that define the range of timescales that can be assessed from the experiment. Using destabilized reporters [51-53,70] would probably improve the precision of our approach towards faster timescales, provided that the fluorescence signal remains sufficiently strong to be detected by flow cytometry. In such cases, it should be possible to resolve burst duration (1/*k_off_*) and transcription rate (*ρ*) separately. Note, however, that having half-lives that are too short could impair the ability to probe long timescales, such as the time between bursts. In addition, resolving experimentally the full distribution of chromatin open/closed times (that is, the distributions of *k_on _*and *k_off_*) is only possible with single-cell time-lapse experiments [51,52].

Finally, using our mathematical model, we simulated a situation in which the chromatin dynamics were directly modified by TSA. As expected, TSA treatment activated the mean reporter-gene expression [26,29,72] and seemed to increase the fraction of time spent in the 'on' phase, probably as a result of a permissive chromatin state [42,50,58]. The direct consequence of this treatment was a gradual shift of the distributions towards higher fluorescence values. After testing several possible chromatin dynamics leading to chromatin opening, our model was able to produce simulated distributions that efficiently fitted the flow-cytometry values during most of the TSA treatment. Moreover, the results suggest that TSA treatment does not increase the duration of the individual 'on' phase, but rather increases the frequency of these phases by reducing the duration of the 'off' phase, thus globally increasing the relative proportion of 'on' phases, and hence increasing the transcriptional activity. It should be notes that, owing to the instantaneous modification of the chromatin dynamics imposed in the model, the simulated distributions were a poor fit to the flow-cytometry data during the first stage of the treatment, whereas they were a perfect fit at the end of the treatment. In order to analyze the kinetics of chromatin opening, a significant improvement of our model would be to perform more precise modeling of treatment kinetics leading the new chromatin dynamics. Our study highlights the importance of chromatin-opening events in the regulation of transcription. It suggests that, to fine-tune the level of expression variability of a gene, higher eukaryotic cells might act on the chromatin mean closed time. This result provides new clues about the mechanisms involved in stochastic gene-expression regulation by chromatin remodeling.

Our work suggests that the probability of chromatin entering an open state is a key determinant of gene expression in our system. A recent study in *Escherichia coli*, using a somewhat different strategy, identified that the *k_off _*parameter (probability of shifting into a transcriptionally closed state) was the main parameter used by the bacterium for gene upregulation [73], which is therefore in sharp contrast to our own results. This might be related to the different biophysical nature of the 'on' and 'off' states in prokaryotes versus eukaryotes, owing to the specific nature of chromatin in eukaryotes. Finally, our results also emphasize the very slow dynamics of chromatin. Indeed, this work suggests that, depending on the genomic location of the transgene, chromatin can stay in a closed state for days, switching only occasionally to an open active state. This emphasizes the slowness of the stochastic-expression process. However, it is important to note that even if chromatin seems to be a major player in regulating gene-expression noise, we did not explore the numerous other possible sources of stochasticity such as cellular division [74,75], elongation dynamics [76], the combinatorial interplay of complexes at the promoter [77], presence of transcription factories [49], and other spatial aspects [78]. Solutions for dissecting the contribution of all the components of the regulation of stochastic gene expression could be found by 1) dedicated experimental studies, as for example in the recent work by Singh *et al*., in which the authors proposed a method to discriminate between mRNA birth/death and promoter fluctuations as intrinsic sources of noise [70], coupled with 2) a progressive increase in the model complexity based on advances in our understanding of the different mechanisms involved in the stochasticity of gene expression.

## Conclusions

In this study, we highlight the importance of the dynamics of chromatin in the control of cell-to-cell variability. Our results suggest that long periods of 'off' time (during which transcription does not occur) followed by brief period of 'open' times (with a strong transcriptional activity) can best explain the observed difference between clones in terms of stochastic gene expression. This paves the way for future studies exploring the role of chromatin dynamics at a more local scale.

## Methods

### Cell culture

All experiments were performed on 6C2 cells, a chicken erythroblast cell line transformed by the avian erythroblastosis virus (*AEV*) [79,80]. Cells were maintained in alpha minimal essential medium (Gibco-BRL,Gaithersburg, MD, USA) supplemented with 10% (v/v) fetal bovine serum, 1% (v/v) normal chicken serum, 100 µmol/l β-mercaptoethanol (Sigma-Aldrich, St Louis, MO, USA), 100 units/ml penicillin and 100 μg/ml streptomycin (Gibco-BRL), at a maximum density of 1 × 10^6 ^cells per ml.

### Generation of stably transfected clones

Stably transfected clones, expressing a fluorescent reporter, were obtained as previously described [81]. Briefly, 6C2 cells were nucleofected in a transfection apparatus (Nucleofector™ II; Amaxa Nucleofector™ Technology) (T-16 program) using a commercial kit (Cell Line Nucleofector® Kit V; Lonza GmBH, Cologne, Germany) and a pT2.CMV-*mCherry*/pCAGGS-T2TP plasmid mix (ratio 5/1). The pT2.CMV-*mCherry *plasmid was constructed using the same strategy as described for the pT2.CMV-*hKO *plasmid [81], except that the *hKO *reporter gene was replaced by *mCherry*, extracted from the pRSET-B plasmid (kindly provided by Dr Roger Tsien, University of California, San Diego, CA, USA). mRNA birth/death fluctuations constitute a major source of stochasticity in gene expression because many mRNA species are present at very low molecular counts within cells [58,70,82,83], thus we reduced this source of intrinsic noise by using the cytomegalovirus (CMV) promoter. Obtaining a strong signal also allowed us to overcome bias caused by autofluorescence in the flow-cytometry data. The integration into genomic DNA of the reporter is allowed by the Tol2 transposon system [84]; the CMV-*mCherry *sequence, flanked by Tol2 motifs, is recognized by a transposase (pCAGGS-T2TP), and randomly inserted into 6C2 genomic DNA. Seven days after transfection, stably transfected cells expressing the reporter gene were sorted and individually cloned in U-shaped 96-well microplates (Cellstar Greiner Bio-One GmBH, Frickenhausen, Germany) using a cytometer (FACSVantage SE; Becton-Dickinson, Franklin Lakes, NJ, USA).

### Molecular and cellular characterization of clones

For each clone, the genomic reporter insertion sites were identified using a splinkerette PCR method as previously described [81], in order to select only clones with a single insertion site. Briefly, genomic DNA isolated from clones expressing the gene reporter was purified by phenol extraction and ethanol precipitation, before being digested for 16 hours at 65°C with *Tai*I, a restriction enzyme with a 4 bp recognition site. The digested DNA was then ligated to a splinkerette adaptor for 1 hour at 22°C. After purification of the ligated product, two rounds of PCR (PCR1 and nested PCR2) were performed using primers specific for the reporter transgene *mCherry *and for the annealed splinkerette adaptor, and a commercial polymerase (AccuPrime™ Taq DNA Polymerase High Fidelity; Invitrogen Inc., Carlsbad, CA, USA). The PCR products were then purified and sequenced. Finally, the genomic reporter insertion sites were identified by similarity searches using the sequence analysis tool iMapper [85]. The identification of the insertion sites of the selected clones was confirmed using a high-throughput splinkerette-PCR method [86], allowing the analyses of hundreds of clones. This work will be described in details elsewhere.

For characterization of clones and analysis of treatment effects (see below), flow-cytometry analyses were performed (FACSCanto II; Becton-Dickinson) on cells extemporaneously pelleted and resuspended in Dulbecco's phosphate-buffered saline 1× solution (Gibco-BRL). Each sample was analyzed using an acquisition of 50,000 events (gated on living cells), and the positive fluorescence threshold was fixed using non-transfected cells. Possible variability resulting from flow-cytometer calibration was taken into account by systematically analyzing flow-calibration particles (SPHERO™ Rainbow; Spherotech Inc., Lake Forest, IL, USA), as a calibration reference.

Non-transfected cells were used to measure 6C2 native autofluorescence, and the difference between the fluorescence of transfected and non-transfected ones was used as an indicator of the transgene activity (note that autofluorescence was also systematically added to the model's output to compute the distribution distance scores).

For each clone, two indicators were systematically used: MFI (mean fluorescence intensity) and NV (the variance divided by the square mean).

For a given cell, the measured fluorescence *f *(from the flow cytometer) is *f *= *f_t _*+ *f_a_*; that is, the sum of the true fluorescence *f_t _* (coming from the reporter proteins) and the autofluorescence *f_a _*(coming from the rest of the cell). The autofluorescence is not a constant, but has a distribution that is obtained using non-transfected cells. The two first moments of *f *read simply as

$$\left\langle f\right\rangle =\left\langle f_{t}\right\rangle +\left\langle f_{a}\right\rangle$$

and

$$\sigma^{2}\left(f\right)=\sigma^{2}\left(f_{t}\right)+\sigma^{2}\left(f_{a}\right).$$

Hence, with MFI and NV being the mean and normalized variance of the true fluorescence, we get:

$$MFI=\left\langle f\right\rangle -\left\langle f_{a}\right\rangle$$

and

$$NV=\frac{\sigma^{2}\left(f\right)-\sigma^{2}\left(f_{a}\right)}{{\left(\left\langle f\right\rangle -\left\langle f_{a}\right\rangle \right)}^{2}}$$

Finally, to compare the theoretical distributions obtained from simulations (which only included the reporter fluorescence) with those obtained from experiments (which also included the autofluorescence), the model's output was first combined with the experimental autofluorescence. This was carried out by summing each simulation result with the value of a randomly selected cell from the autofluorescence distribution. The resulting distribution was the convolution between the theoretical and the autofluorescence distributions, and was then compared with the experimental distributions using a Kolmogorov-Smirnov test.

### Determination of *mCherry *mRNA and protein degradation rates

To determine the *mCherry *mRNA degradation rate, the mRNA concentration was estimated using quantitative reverse transcription (qRT)-PCR after transcription inactivation was achieved using actinomycin D treatment. Two clones (C5 and C11) were treated, in duplicate, for 0, 60, 124, 244 and 488 minutes with a final concentration of 10 µg/ml actinomycin D (A9415; Sigma-Aldrich), before extracting the mRNA after the instructions of RNeasy® Plus Mini Kit (Qiagen Inc., Valencia, CA, USA). To prepare the real-time PCR assay, 1 µg of total RNA from each sample was reversed transcribed using the SuperScript^™ ^III First-Strand Synthesis System for RT-PCR (Invitrogen Inc.) in the presence of random hexamers. Quantification of mRNA levels by real-time PCR was performed in 96-well plates using a real-time PCR system (LightCycler 480; Roche Diagnostics, Basel, Switzerland). The measurement was performed in a final volume of 10 µl of reaction mixture (containing 2.5 µl of cDNA template diluted 1 in 5), prepared using a commercial kit (Light Cycler 480 SYBR Green I Kit; Roche Diagnostics) in accordance with the manufacturer's instructions, and with the primer set at a final concentration of 0.5 µmol/l (mCher-For: CCACCTACAAGGCCAAGAA, mCher-Rev: ACTTGTACAGCTCGTCCATG). An internal standard curve was generated using serial dilutions (from 2000 to 0.02 fg/µl) of purified PCR product. The reactions were initiated by activation of *Taq *DNA polymerase at 95°C for 5 minutes, followed by 45 three-step amplification cycles consisting of denaturation at 95°C for 15 seconds, annealing at 55°C for 15 seconds, and extension at 72°C for 15 seconds. The fluorescence signal was measured at the end of each extension step. After the amplification, a dissociation stage was run to generate a melting curve for verification of amplification-product specificity. The crossing point (CP) was determined by the second derivative maximum method in the LightCycler® 480 software (version 1.5.0). After normalization, taking into account cellular viability and mRNA quantity used for the retrotranscription step, the mRNA half-life was determined by fitting mRNA quantity evolution by a decreased exponential (least square) method.

To determine the mCherry protein degradation rate, we used flow cytometry to measure the protein half-life after translation inactivation using cycloheximide treatment. C5 and C11 clones were treated in duplicate for 0, 16, and 24 hours with a final concentration of 100 µg/µl cycloheximide (C4859; Sigma-Aldrich), and for each time point, the fluorescence of the treated cells was measured by flow cytometry. The autofluorescence component was removed as explained earlier. The protein half-life was determined using exponential fit of the fluorescence mean decrease curve, similarly to the procedure used for determining the mRNA half-life.

### Treatments with chromatin-modifying agents

To analyze the effect of chromatin state on the stochasticity of gene expression, clones were treated with TSA, a histone deacetylase inhibitor (P5026; Sigma-Aldrich) and 5-AzaC, an inhibitor of DNA methylation (A2385; Sigma-Aldrich). For each clone, kinetic treatment experiments were performed; clones were treated with 500 nmol/l TSA or 500 µmol/l 5-AzaC at five time points (0, 8, 24, 32, and 48 hours). For each time point, 1 × 10^6 ^cells (for 0, 8, and 24 hours) or 5 × 10^5 ^cells (for 32 and 48 hours) were treated with the relevant drug and characterized by flow cytometry.

### Model description

The two-state model of gene expression represents the chromatin activity as an 'on-off' process specified through the transition rates *k_on _*and *k_off _*(respectively representing the 'off-on' transition and the 'on-off' transition). To enable comparison with the experimental data, a simple model of mRNA and protein dynamics based on two production/degradation models completed the model. The production of mRNA was allowed only in the 'on' state (open chromatin) but completely forbidden in the 'off' state (closed chromatin). The model thus corresponds to the following equations:

(1) $$\left\{\begin{array}{l} k_{T}=\frac{k_{on}}{k_{on}+k_{off}}\\ \frac{dR}{dt}=\rho k_{T}-\overset{˜}{\rho }R\\ \frac{dP}{dt}=\gamma R-\overset{\sim }{\gamma }P\\ f=\alpha P \end{array}\right)$$

where*,k_on _*is the closed-to-open transition rate, *k_off _*is the open-to-closed transition rate, and *k_T _*is the resulting proportion of the 'on' state; *R *is the number of mRNAs, *ρ *is the mRNA production rate (when chromatin is open), and $\tilde{\rho }$ is the mRNA degradation rate; *P *is the number of mCherry proteins, *γ *is the mCherry production rate (per mRNA) and $\tilde{\gamma }$ is the mCherry degradation rate; *f *is the fluorescence intensity of the cell (after subtraction of the autofluorescence) and *α*, is a linear proportionality coefficient to convert the number of proteins into arbitrary fluorescence measures.

This model can be simulated with the SSA (see below) to ascertain the behavior of single cells and eventually to compute the fluorescence distributions. It can also be analytically derived to compute the MFI and NV of large cell populations at steady state.

### Analytical derivation of the model

Paulsson proposed an analytic expression of the mean quantity and NV of protein in the two-state model, as a function of chromatin-dynamics parameters and transcription-translation parameters [45]. In the case of a single gene and taking into account the parameter *α*, Paulsson's equation gives:

(2) $$\left\{\begin{array}{l} MFI=\alpha \;\frac{\rho \;\gamma }{\overset{\sim }{\rho }\;\overset{\sim }{\gamma }}\frac{k_{on}}{k_{on}+k_{off}}\\ NV=\left(\frac{\overset{\sim }{\rho }\;\overset{\sim }{\gamma }}{\rho \;\gamma }\;\frac{k_{on}+k_{off}}{k_{on}}\right)+\left(\frac{\overset{\sim }{\rho }}{\rho }\;\frac{k_{on}+k_{off}}{k_{on}}\;\frac{\overset{\sim }{\gamma }}{\overset{\sim }{\rho }+\overset{\sim }{\gamma }}\right)+\left(\frac{k_{off}}{k_{on}}\;\frac{\overset{\sim }{\gamma }}{\overset{\sim }{\rho }+\overset{\sim }{\gamma }}\;\frac{1+\frac{\overset{\sim }{\rho }}{\overset{\sim }{\gamma }+k_{on}+k_{off}}}{1+\frac{k_{on}+k_{off}}{\overset{\sim }{\rho }}}\right) \end{array}\right)$$

This equation can be used to express *k_on _*and *k_off _* as a function of MFI, NV, and the transcription-translation parameter sets. Rewriting the equation gives:

(3) $$\left\{\begin{array}{c} A=\alpha \frac{\rho \;\gamma }{MFI\;\tilde{\rho }\;\tilde{\gamma }}\\ B=\frac{A\frac{\overset{\sim }{\rho }}{\rho }\left(\frac{\tilde{\gamma }}{\gamma }+\frac{\overset{\sim }{\gamma }}{\overset{\sim }{\rho }+\overset{\sim }{\gamma }}\right)-NV}{\left(A-1\right)\;\frac{\overset{\sim }{\gamma }}{\overset{\sim }{\rho }+\overset{\sim }{\gamma }}}\\ {\left(A\left(B\left(\overset{\sim }{\gamma }+\overset{\sim }{\rho }\right)+\overset{\sim }{\rho }\right)\right)}^{2}-4A^{2}B\overset{\sim }{\rho }\left(\overset{\sim }{\rho }+\overset{\sim }{\gamma }\left(B+1\right)\right)\;>0\\ k_{on}=\frac{-A\left(B\left(\overset{\sim }{\gamma }+\overset{\sim }{\rho }\right)+\overset{\sim }{\rho }\right)-\sqrt{{\left(A\left(B\left(\overset{\sim }{\gamma }+\overset{\sim }{\rho }\right)+\overset{\sim }{\rho }\right)\right)}^{2}-4A^{2}B\overset{\sim }{\rho }\left(\overset{\sim }{\rho }+\overset{\sim }{\gamma }\left(B+1\right)\right)\;}}{2A^{2}B}\\ k_{on}>0\\ k_{off}=\left(A-1\right)k_{on} \end{array}\right)$$

### Parametric exploration of the analytical model

Because the clonal populations differed only in their insertion points (that is, their chromatin-dynamics parameters), equation 3 enabled us to find the clone-specific parameters from MFI and NV (measured by flow cytometry) and the transcription-translation parameters *ρ*, $\tilde{\rho }$, *γ*, $\tilde{\gamma }$ and *α*.$\tilde{\rho }$ and $\tilde{\gamma }$ can be determined experimentally (see above) but *ρ*, *γ *and *α *remained unknown. We explored a wide range of these parameters (large enough to include all biologically relevant values): *ρ *= 6.0, 1.0, 0.5, 0.333, 0.25, 0.200, 0.1666, 0.14286, 0.125, 0.111, 0.100, 0.0500, 0.0333, 0.0250, 0.02, 0.01666, 0.01333, 0.0111, 0.00952, and 0.00833 mRNA/min, corresponding to one mRNA produced each 1/*ρ *= 10 seconds, 1, 2, 3, 4, 5, 6, 7, 8, 9, 10, 20, 30, 40, and 50 minutes, and 1, 1.25, 1.5, 1.75, and 2 hours, when chromatin is in the open state; *γ *= 1.0, 0.200, 0.100, 0.0333, 0.01667, 0.006667, 0.00333, 0.00222, 0.001666, 0.001333, 0.00111, 0.0008333, 0.00069444, 0.00046296, and 0.0003472 protein/min/mRNA, corresponding to a protein produced each 1/*γ *= 1, 5, 10, and 30 minutes, 1, 2.5, 5, 7.5, 10, 12.5, 15, and 20 hours, and 1, 1.5 and 2 days per mRNA molecule; and *α *= 0.10, 0.15, 0.50, 1.0, 1.5, 5.0, 10.0, 15.0, 50.0, 100.0, 150.0, and 200.0 arbitrary units.

Exploring all values of *ρ*, *γ *and *α *gave us 3,600 couples (*k_on_*; *k_off_*), of which only 1,047 respected the condition mentioned in equation 3 (*k_on_*>0).

### Comparison between the analytical model and the trichostatin A-treated clones

Equation 1 enabled us to compute the mean mRNA number (*R*) and the mean protein number (*P*) at steady state, from the values of the chromatin-dynamics and transcription-translation parameters:

(4) $$\left\{\begin{array}{c} R=\frac{\rho }{\overset{\sim }{\rho }}\left(\frac{k_{on}}{k_{on}+k_{off}}\right)\\ P=\left(\frac{\rho }{\overset{\sim }{\rho }}\;\frac{\gamma }{\overset{\sim }{\gamma }}\right)\left(\frac{k_{on}}{k_{on}+k_{off}}\right) \end{array}\right)$$

Then, assuming that at *t *= 0, the cell switches to a new chromatin dynamics (because of the TSA treatment), compute *R*(*t*), (the evolution of mRNA number), and *P*(*t*), (the evolution of protein number following the TSA treatment) can be computed. If $k_{on}^{TSA}$ and $k_{off}^{TSA}$ are the new chromatin-dynamics parameters induced by the treatment, the equation is:

(5) $$\left\{\begin{array}{c} R\left(t\right)=\frac{\rho }{\overset{\sim }{\rho }}\left(\frac{k_{on}}{k_{on}+k_{off}}-\frac{k_{on}^{TSA}}{k_{on}^{TSA}+k_{off}^{TSA}}\right)e^{-\overset{\sim }{\rho }t}+\frac{k_{on}^{TSA}}{k_{on}^{TSA}+k_{off}^{TSA}}\frac{\rho }{\overset{\sim }{\rho }}\\ P\left(t\right)=\left(\frac{\rho }{\overset{\sim }{\rho }}\frac{\gamma }{\overset{\sim }{\gamma }}-\frac{\rho }{\overset{\sim }{\rho }}\;\frac{\gamma }{\left(\overset{\sim }{\gamma }-\overset{\sim }{\rho }\right)}\right)\left(\frac{k_{on}}{k_{on}+k_{off}}-\frac{k_{on}^{TSA}}{k_{on}^{TSA}+k_{off}^{TSA}}\right)e^{-\overset{\sim }{\gamma }t}\\ \;\;\;\;\;\;+\frac{\rho }{\overset{\sim }{\rho }}\frac{\gamma }{\left(\overset{\sim }{\gamma }-\overset{\sim }{\rho }\right)}\left(\frac{k_{on}}{k_{on}+k_{off}}-\frac{k_{on}^{TSA}}{k_{on}^{TSA}+k_{off}^{TSA}}\right)e^{-\overset{\sim }{\rho }t}+\frac{\rho }{\overset{\sim }{\rho }}\frac{\gamma }{\overset{\sim }{\gamma }}\;\frac{k_{on}^{TSA}}{k_{on}^{TSA}+k_{off}^{TSA}} \end{array}\right)$$

The exact values of $k_{on}^{TSA}$ and $k_{off}^{TSA}$ remained unknown at this stage, but we could simulate the extreme situation by assuming that, under TSA treatment, the chromatin is fully open. Analytically, this gives:

(6) $$\frac{k_{on}^{TSA}}{k_{on}^{TSA}+k_{off}^{TSA}}=1$$

Note that this equation represents an extreme situation, not the exact TSA influence on chromatin.

Introducing equation 6 into the dynamics of equation 5, we were able to compute, for a given transcription-translation parameter set, the maximum rate of protein concentration increase, and thus the maximum increase of reporter fluorescence. For each parameter set, we compared the predicted fluorescence increase under the extreme condition of a fully open chromatin. We then rejected all parameter sets for which the protein number did not increase sufficiently rapidly to account for the fluorescence increase measured experimentally during TSA treatment.

### Simulation of the model

The model can be simulated using an SSA, which is an exact continuous-time algorithm that enables simulation of chemical-reaction systems [54]. Each simulation represents one of the possible realizations of the system from a specified initial state and for a given kinetic parameter set (these parameters being here considered as probabilities). Each realization depends on a pseudo-random generator, and different realizations (that is, simulations of different cells issued from the same clone) can be computed by simply initializing this random generator with different seeds. The implementation of the two-state model (equation 1) in the SSA enables simulation of the entire system dynamics and visualization of the course of chromatin state, gene transcription, number of mRNAs, mRNA translation, number of proteins and, ultimately fluorescence, in a virtual single cell. By simulating a large number of such 'artificial cells', we were able to simulate 'virtual flow-cytometry experiments' and to compute MFI, NV, and full distribution for a given parameter set. We simulated 50,000 virtual cells for 30,000 minutes (a sufficiently long period to ensure that all cells were at a steady state, the concentration values being initialized at the theoretical values given by the analytical model). The fluorescence of each cell was then computed, and the simulated distribution generated through convolution with the autofluorescence of the 6C2 cells measured experimentally (see above). Simulated distributions were then compared with the experimental distribution using the Kolmogorov-Smirnov test. The quality of each parameter set was then evaluated (the score of a given parameter set being the mean Kolmogorov-Smirnov score of each clone). The best parameter set was thus the one that gave the best fit for all six clonal populations.

### Simulation of trichostatin A treatment in the model

Using the best parameter set, we simulated 50,000 cells of the two TSA-treated clones C5 and C11 for 30,000 minutes. The chromatin-dynamics parameters were then modified to account for the TSA treatment, and the two clones were simulated for a further 1,152 minutes (48 hours). For each clones, the simulated distributions were computed after 8, 24, 32 and 48 hours, and compared with the experimental distributions using a Kolmogorov-Smirnov test. The best chromatin-dynamics parameters ($k_{on}^{TSA}$; $k_{off}^{TSA}$) were those that gave the best mean score at the four time points. In total, 11 different chromatin-dynamics values were tested for each clone. Note that, knowing the MFI value of the treated clones, we could analytically compute the $k_{T}^{TSA}$ value (using equation 5). Taking

$$k_{T}^{TSA}=\frac{k_{on}^{TSA}}{k_{on}^{TSA}+k_{off}^{TSA}}$$

(from equation 1), we can use this analytical value to simplify the parametric exploration.

## Competing interests

The authors declare that they have no competing interests.

## Authors' contributions

JV, GK, AC, GB, and OG conceived and designed the research. JV performed all the biological experiments. GK performed all the computational analyses and model simulations. AC provided assistance for the modeling analyses. EV and VM provided technical assistance for the biological experiments. CMP and JJK advised on the design and interpretation of the experiments. JV, GK, AC, GB, and OG wrote the paper. OG and GB co-supervised the project. All authors have read and approved the final manuscript.

## Supplementary Material

Additional file 1 **Table S1. Identification by splinkerette PCR of the *mCherry *genomic insertion sites for six 6C2 cellular clones**. Click here for file

Additional file 2 **Figure S1. Determination of the *mCherry *reporter mRNA and protein half-lives**. **(A) **Quantitative reverse transcription PCR measurement of *mCherry *mRNA decay after actinomycin D treatment in two different clones of the 6C2 cell line. The best-fitting exponential curve (black line) was found by minimizing least squares (between exponential curve and biological data). The deduced *mCherry *mRNA half-life was 7 hours and 4 minutes (424 minutes). **(B) **Flow-cytometry measurement of mCherry protein fluorescence decay after cycloheximide treatment in two different clones of the 6C2 cell line. The best-fitting exponential curve (black line) was found by minimizing least squares (between exponential curve and biological data). The deduced mCherry protein half-life was 65 hours and 47 minutes (3,947 minutes). For both parts, ordinates are on a logarithmic scale. Click here for file

Additional file 3 **Figure S2. Exploration of model parameters based on a comparison of fluorescence distributions and SSA simulations**. This figure is similar to the Figure 4 except that the selected parameter set had the highest (that is, worst) score (shown as a brown circle in the upper left part of the figure) of the best scores obtained. Click here for file

Additional file 4 **Table S2. *mCherry *transcription rates and mRNA levels for six cellular clones of the 6C2 cell line**. Click here for file

Additional file 5 **Figure S3. Model simulation of the perturbation of chromatin dynamics by TSA treatment**. This figure is similar to the Figure 6 except that the best new chromatin dynamics was computed from the parameter set which had the highest (that is, worst) score (shown as a brown circle in the panel (A) of Figure S2 in Additional file 3) of the best scores obtained. Click here for file
