# Supplementary material for: Quantifying the contribution of chromatin dynamics to stochastic gene expression reveals long, locus-dependent periods between transcriptional bursts
Source: BMC Biol. 2013 Feb 25;11:15. doi: 10.1186/1741-7007-11-15 (PMC3635915; doi:10.1186/1741-7007-11-15)
Supplement: Additional file 1 — Table S1. Identification by splinkerette PCR of the mCherry genomic insertion sites for six 6C2 cellular clones. [file 1741-7007-11-15-S1.PDF]

Additional file 1

Table S1

Identification by splinkerette PCR of the *mCherry* genomic insertion sites for six 6C2 cellular clones.

Clone	Chromosome	Chromosomic location	Direction
C1	Z	54910770	Reverse
C3	2	145804848	Forward
C5	15	1558243	Forward
C7	2	146026983	Forward
C11	11	12399458	Reverse
C17	2	145558788	Reverse
