# Supplementary figures and images for: Quantifying the contribution of chromatin dynamics to stochastic gene expression reveals long, locus-dependent periods between transcriptional bursts
Source: BMC Biol. 2013 Feb 25;11:15. doi: 10.1186/1741-7007-11-15 (PMC3635915; doi:10.1186/1741-7007-11-15)

## Additional file 2

**A**

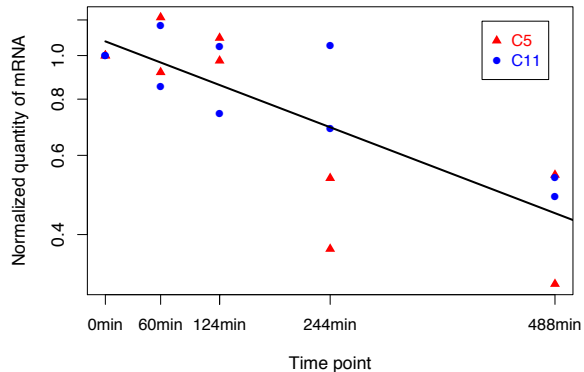

**B**

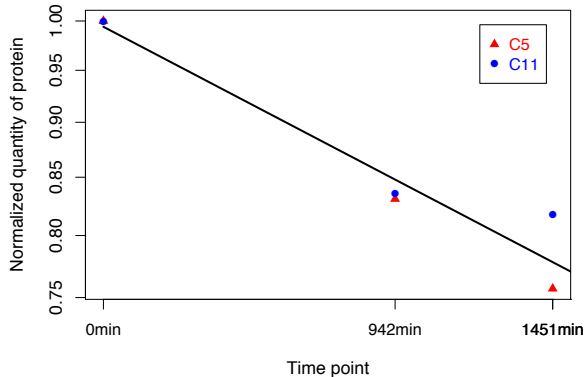

Supplement: Additional file 2 — Figure S1. Determination of the mCherry reporter mRNA and protein half-lives. (A) Quantitative reverse transcription PCR measurement of mCherry mRNA decay after actinomycin D treatment in two different clones of the 6C2 cell line. The best-fitting exponential curve (black line) was found by minimizing least squares (between exponential curve and biological data). The deduced mCherry mRNA half-life was 7 hours and 4 minutes (424 minutes). (B) Flow-cytometry measurement of mCherry protein fluorescence decay after cycloheximide treatment in two different clones of the 6C2 cell line. The best-fitting exponential curve (black line) was found by minimizing least squares (between exponential curve and biological data). The deduced mCherry protein half-life was 65 hours and 47 minutes (3,947 minutes). For both parts, ordinates are on a logarithmic scale. [file 1741-7007-11-15-S2.PDF]

Additional file 3

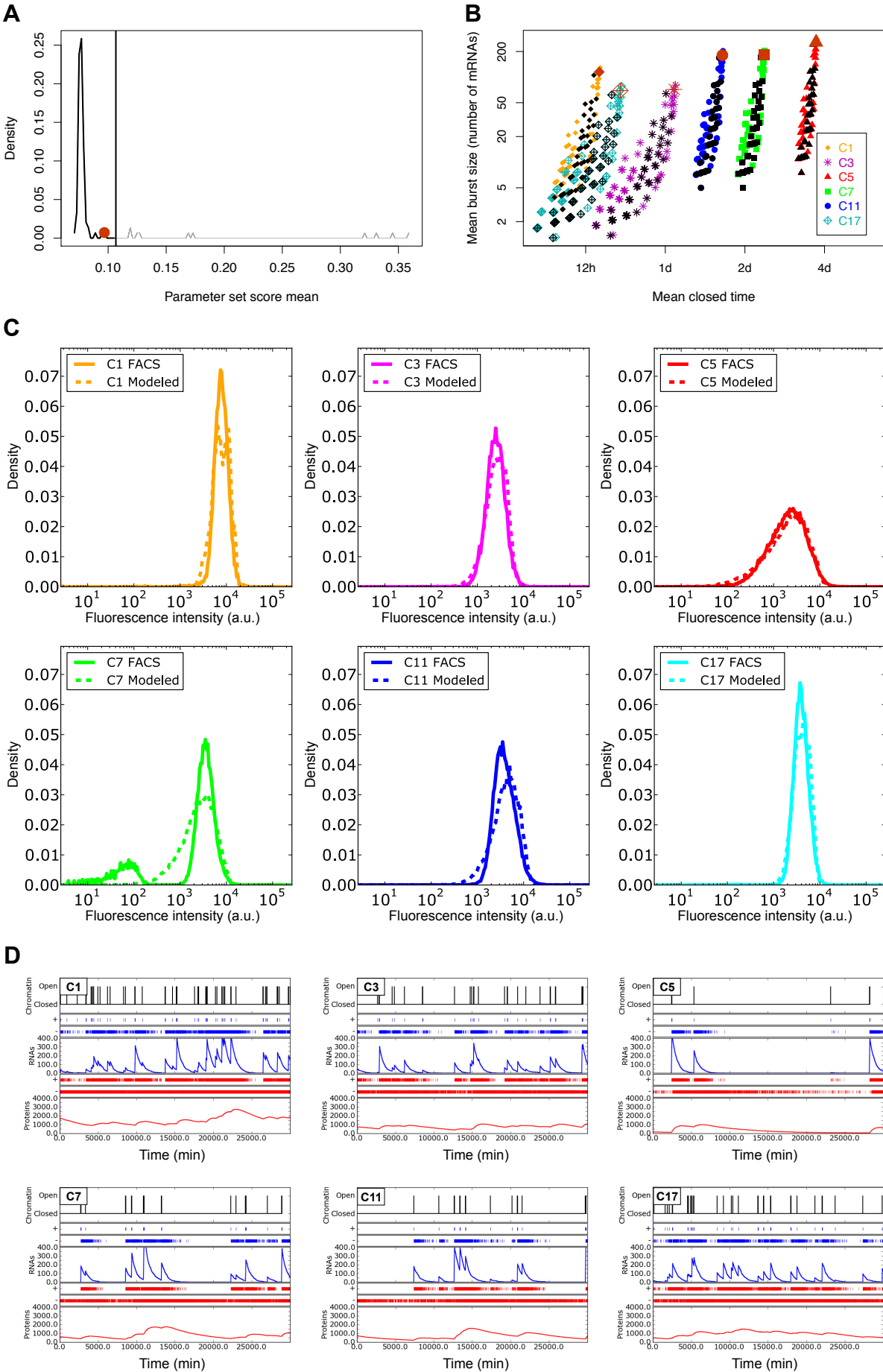

Supplement: Additional file 3 — Figure S2. Exploration of model parameters based on a comparison of fluorescence distributions and SSA simulations. This figure is similar to the Figure 4 except that the selected parameter set had the highest (that is, worst) score (shown as a brown circle in the upper left part of the figure) of the best scores obtained. [file 1741-7007-11-15-S3.PDF]

Additional file 5

A

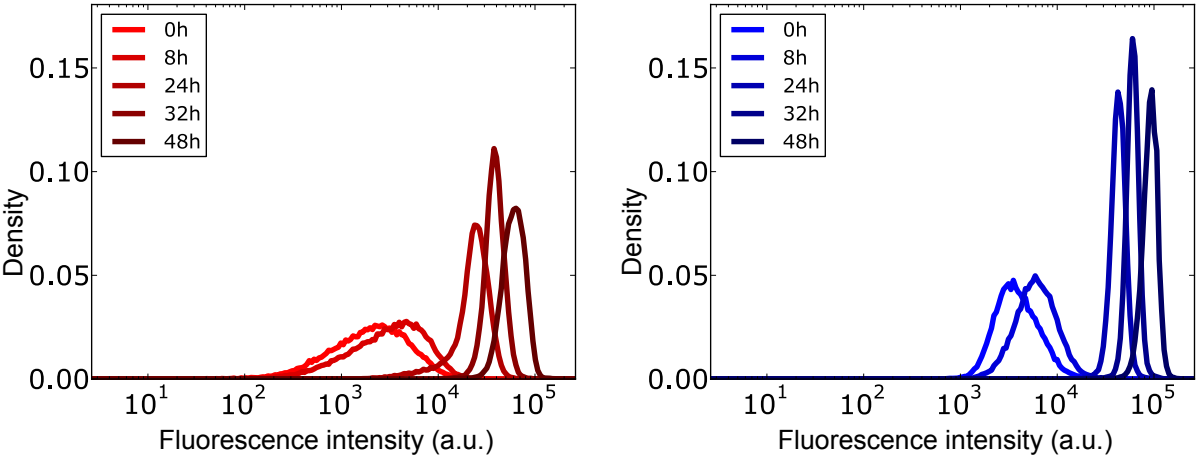

B

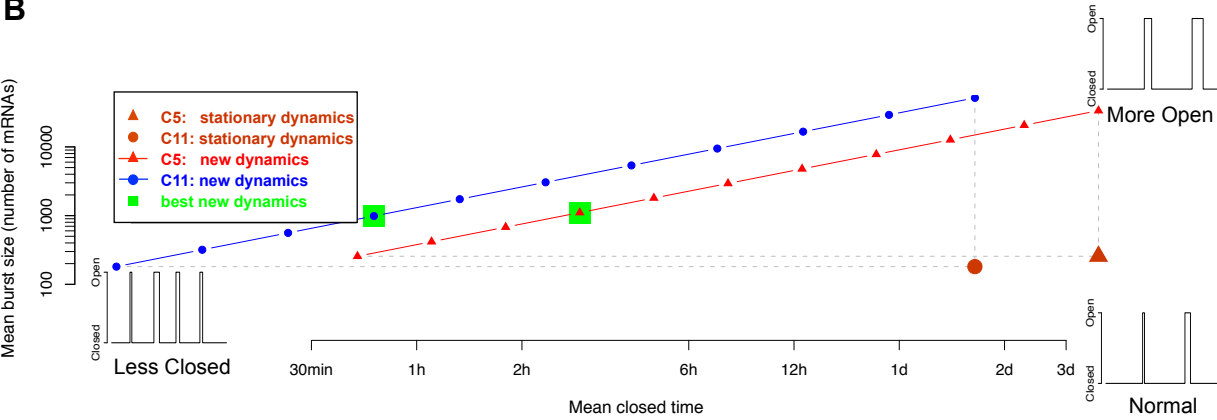

C

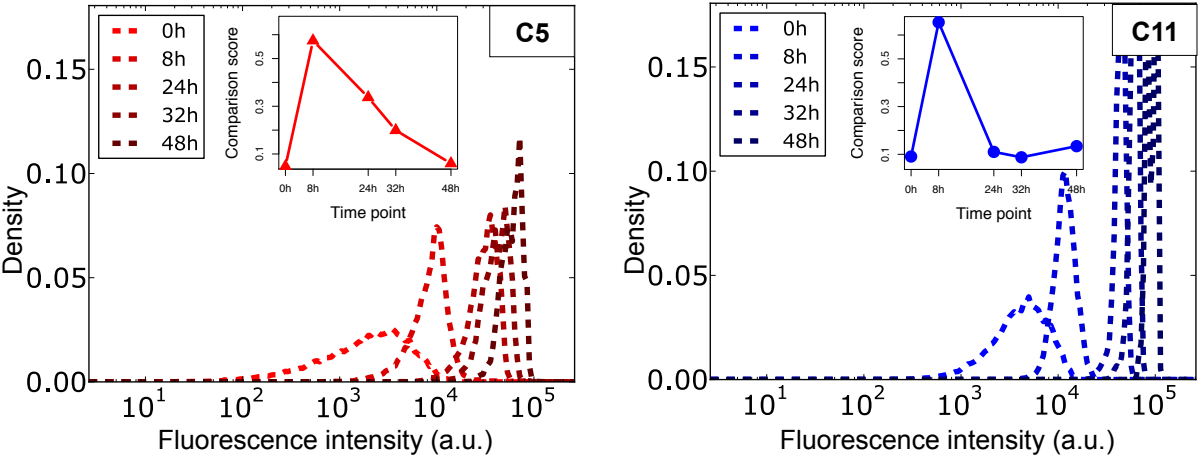

D

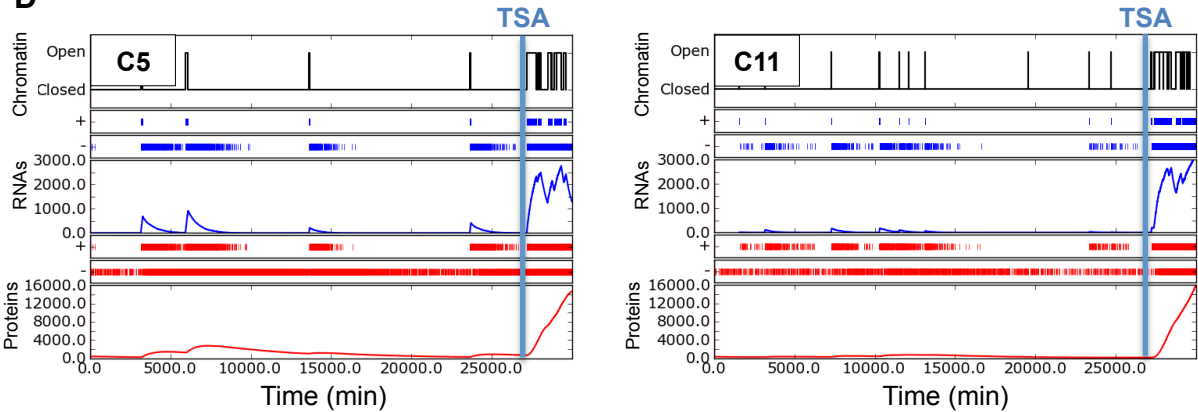

Supplement: Additional file 5 — Figure S3. Model simulation of the perturbation of chromatin dynamics by TSA treatment. This figure is similar to the Figure 6 except that the best new chromatin dynamics was computed from the parameter set which had the highest (that is, worst) score (shown as a brown circle in the panel (A) of Figure S2 in Additional file 3) of the best scores obtained. [file 1741-7007-11-15-S5.PDF]
