# Supplementary material for: Quantifying the contribution of chromatin dynamics to stochastic gene expression reveals long, locus-dependent periods between transcriptional bursts
Source: BMC Biol. 2013 Feb 25;11:15. doi: 10.1186/1741-7007-11-15 (PMC3635915; doi:10.1186/1741-7007-11-15)
Supplement: Additional file 4 — Table S2. mCherry transcription rates and mRNA levels for six cellular clones of the 6C2 cell line. [file 1741-7007-11-15-S4.PDF]

Additional file 4

Table S2

mCherry transcription rates and mRNA levels for six cellular clones of the 6C2 cell line.

Clone	mRNA transcription rate [a]	mRNA copies per cell [b]
C1	3.99	40.76
C3	1.31	13.41
C5	1.37	13.95
C7	1.53	15.60
C11	2.17	22.17
C17	2.03	20.72

[a.] mRNA transcription rates are expressed as $mRNA.h^{-1}$ and calculated for each clone as follow : $60^* \rho^*(k_{on}/(k_{on}+k_{off}))$.

[b.] mRNA copies per cell are calculated by dividing mRNA transcription rate by mRNA degradation rate.
